# *MALAT1*/*miR-146a*/COX-2 Expression Profile Six Months After Myocardial Infarction and Association of *MALAT1* rs3200401 and *miR-146a* rs2910164 with Disease Susceptibility

**DOI:** 10.3390/biomedicines14071433

**Published:** 2026-06-24

**Authors:** Natasa Macak Stefanovic, Tamara Djuric, Ivana Kolic, Milica Dekleva, Goran Stankovic, Maja Zivkovic, Ana Djordjevic

**Affiliations:** 1 Laboratory for Radiobiology and Molecular Genetics, VINČA Institute of Nuclear Sciences—National Institute of the Republic of Serbia, University of Belgrade, 11000 Belgrade, Serbia; natasa.macak@vin.bg.ac.rs (N.M.S.); tamariska@vin.bg.ac.rs (T.D.); ivanak@vin.bg.ac.rs (I.K.); majaz@vin.bg.ac.rs (M.Z.); 2 Faculty of Medicine, University of Belgrade, 11000 Belgrade, Serbia; dekleva.milica@gmail.com (M.D.); goranstan@gmail.com (G.S.); 3 Department of Cardiology, Clinical Centre of Serbia, 11000 Belgrade, Serbia

**Keywords:** six months post-myocardial infarction, lncRNA MALAT1, miR-146a, MALAT1/miR-146a/COX-2 axis, COX-2, expression, ELISA, rs3200401, rs2910164

## Abstract

**Background/Objectives:** Inflammatory and oxidative-stress-related processes contribute to post-myocardial infarction (MI) remodeling and may influence long-term cardiovascular outcomes. Recent findings have highlighted the potential role of non-coding RNAs in regulating these processes. LncRNA MALAT1 acts as a ceRNA that “sponges” miR-146a, reducing its ability to repress downstream targets such as COX-2. The aim of this study was to assess MALAT1 and miR-146a expression in PBMCs and plasma COX-2 in controls and patients six months post-MI. In addition, we investigated whether *MALAT1* rs3200401 and *miR-146a* rs2910164 variants were associated with MI susceptibility, MALAT1 and miR-146a expression, plasma COX-2 levels, and left ventricle (LV) echocardiographic parameters. **Methods:** The study included 534 patients and 381 controls for genetic analyses, while expression analyses were performed in a subset of 89 patients and 39 controls. TaqMan™ assays were used for genotyping and for quantification of MALAT1 and miR-146a expression. Plasma COX-2 levels were measured using ELISA. **Results:** Compared to controls, patients had higher MALAT1 expression, whereas lower miR-146a expression was observed only in unadjusted analyses. Plasma COX-2 levels were higher in patients with advanced heart failure (NYHA III–IV) compared with NYHA I-II. The rs3200401 TT genotype was more frequent in patients, whereas rs2910164 genotype distributions were similar between groups. The rs3200401-rs2910164 TG allele combination was associated with increased MI risk. **Conclusions:** MALAT1 may serve as a potential long-term biomarker of post-MI molecular alterations, whereas the role of miR-146a requires further investigation in larger cohorts. The rs3200401 variant may represent a genetic marker associated with MI susceptibility and adverse LV remodeling. Further studies are needed for confirmation.

## 1. Introduction

With 19.8 million deaths recorded in 2022, according to a new report by the World Health Organization, cardiovascular diseases (CVDs) are the world’s leading cause of death and disability [1]. In most cases, atherosclerosis is the underlying cause of various life-threatening cardiovascular events such as myocardial infarction (MI) and cerebrovascular insult, which together account for approximately 85% of CVD-related deaths. Myocardial infarction initiates a prolonged cascade of molecular and cellular events that extend well beyond the acute, early-stage ischemia, driving chronic inflammation, oxidative stress, chronic late-stage ventricular remodeling and eventually progressive ventricular dysfunction and heart failure (HF) [2]. Emerging evidence suggests that ferroptosis, an iron-dependent form of regulated cell death, may contribute to myocardial injury and adverse cardiac remodeling through mechanisms involving iron dysregulation and lipid peroxidation [3,4,5,6]. Recent findings have highlighted the potential role of non-coding RNAs, including long non-coding RNAs (lncRNAs) and microRNAs (miRNAs), as novel regulators of inflammatory and oxidative-stress-related processes in CVD [7,8,9].

LncRNA MALAT1 (metastasis-associated lung adenocarcinoma transcript 1) is highly expressed in vascular endothelial cells, smooth muscle cells, and cardiomyocytes, where it regulates a key processes involved in atherosclerosis. It plays a role in endothelial cell proliferation and migration, angiogenesis, cardiac fibrosis after MI, endothelial dysfunction, and atherosclerosis-related inflammation and modulates hypoxia-induced endothelial cell injury [10,11,12,13,14]. In human studies, circulating MALAT1 expression was significantly higher in patients with acute MI compared to healthy controls and patients with stable angina, as well as in patients with ST-elevation myocardial infarction (STEMI) who developed the no-reflow phenomenon or major adverse cardiovascular events (MACEs) [15,16,17,18]. MALAT1 acts as a competing endogenous RNA (ceRNA) that “sponges” several miRNAs, including miR-146a, reducing its bioavailability and leading to de-repression of miR-146a targets, such as cyclooxygenase-2 (COX-2) and components of NF-κB signaling [19,20]. Importantly, miR-146a is a key immune-regulatory miRNA highly expressed in circulating immune cells. Given the central role of systemic immune activation in post-MI remodeling, both MALAT1 and miR-146a may be relevant in peripheral blood mononuclear cells (PBMCs), which can reflect systemic inflammatory and oxidative-stress-related processes. COX-2, encoded by *PTGS2*, catalyzes the conversion of arachidonic acid to prostaglandins and thus plays a key role in regulating inflammatory processes [21]. Circulating COX-2 levels have been upregulated in adverse phenotypes, including non-ischemic HF [22]. COX-2 expression has also been associated with lipid peroxidation and inflammatory signaling in myocardial and vascular tissues [23,24]. Recent study has revealed a significant regulatory link between the lncRNA MALAT1, miR-146a, and COX-2, in relation to inflammatory lung disease, chronic obstructive pulmonary disease (COPD) [19].

Despite all the above-mentioned data, limited information is available regarding the expression patterns of MALAT1, miR-146a, and plasma COX-2 levels during the chronic phase following MI. The first six months after MI represent a critical period of post-infarction healing and left ventricular (LV) remodeling, during which inflammatory, fibrotic, and oxidative-stress-related processes continue to evolve and may influence long-term cardiac function and clinical outcomes. By six months, the acute inflammatory response has largely resolved, making this time point appropriate for evaluating persistent molecular alterations that may reflect ongoing post-MI remodeling rather than acute ischemic injury [25,26,27]. We therefore hypothesized that altered expression of these molecules six months after MI may reflect ongoing dysregulation of inflammatory and oxidative-stress-related pathways during the chronic phase following MI. These molecules may therefore represent candidate biomarkers associated with chronic post-MI remodeling and residual myocardial injury.

In addition, we investigated potentially functional variants within MALAT1 and miR-146a, selected based on their reported regulatory potential, the literature evidence, and/or publicly available databases (FIVEx, GTEx, RegulomeDB, ENSEMBLE, HaploReg, GWAS Catalogue, GnomAD). Genetic variability within pathway-related genes may provide additional insight into the regulation of this pathway in the context of myocardial infarction. Accordingly, we selected two gene variants: *MALAT1* rs3200401 and *miR-146a* rs2910164. Variant *MALAT1* rs3200401 is proposed to exert eQTL effects in the blood and cardiovascular tissues, according to the FIVEx and GTEx databases [28,29], and has previously been associated with major adverse cardiovascular and cerebrovascular events (MACCE) and total cholesterol (TC) levels [30,31,32]. It has also been investigated in MI and ischemic stroke (IS), although the available studies have reported conflicting results [31,33,34]. The *miR-146a* rs2910164 variant is a G to C substitution in the stem region of pre-miR-146a. This variant has been shown to affect the processing and expression of miR-146a, although the reported findings regarding its effects remain inconsistent. Jazdzewski et al. reported that the G to C change leads to depletion of miR-146a levels and consequently higher expression of its target genes [35]. A recent meta-analysis found a significant association between *miR-146a* rs2910164 and susceptibility to coronary artery disease (CAD) [36]; however, the participants in these studies were predominantly of Asian ethnicity. Studies on the association of investigated variants with CVDs in Caucasian/European populations are quite limited.

In light of the above, the aim of the present study was to evaluate the expression profile of the MALAT1/miR-146a/COX-2 signaling axis in PBMCs and/or plasma from controls and patients six months after MI. In addition, we investigated the possible association of *MALAT1* rs3200401 and *miR-146a* rs2910164 with MI susceptibility, expression levels of MALAT1 and miR-146a, plasma COX-2 levels and LV echocardiographic parameters, which are crucial for diagnosing and managing conditions such as systolic dysfunction and ischemic HF.

## 2. Materials and Methods

### 2.1. Study Population

A total of 534 patients with a history of the first acute MI and 381 healthy controls were enrolled in the study. All participants were unrelated individuals of Serbian Caucasian origin. The patients were recruited from the Cardiology Clinic, University Clinical Centre of Serbia, Belgrade, Serbia, between March 2024 and December 2025, following admission for the first MI attributable to coronary artery disease (CAD) [37] and subsequent referral for primary percutaneous coronary intervention (PCI). Eligibility criteria excluded individuals older than 70 years, as well as those with a prior history of MI or other cardiac conditions, including congenital or valvular heart disease, cardiomyopathies, or the presence of cardiac devices such as pacemakers or implantable cardioverter–defibrillator. Patients with inadequate echocardiographic imaging or inability to complete required assessments were also excluded. All included patients had angiographically confirmed coronary stenosis exceeding 70% in the infarct-related artery, irrespective of single- or multivessel disease. Standard biochemical analyses were performed at admission using routine laboratory protocols. Hypertension was defined as systolic blood pressure ≥ 140 mmHg, diastolic blood pressure ≥ 90 mmHg, or ongoing antihypertensive therapy. Type 2 diabetes mellitus (T2DM) was defined based on fasting plasma glucose > 7.0 mmol/L or the use of insulin or oral hypoglycemic agents. Control subjects were selected among healthy volunteers without evidence of renal failure, T2DM, chronic inflammatory conditions, or established cardiovascular or cerebrovascular disease.

All participants provided written informed consent prior to inclusion. The study protocol was approved by the Ethics Committee of the University Clinical Centre of Serbia (No 936/16; approval date: 29 February 2024).

### 2.2. Doppler Echocardiography

Doppler echocardiographic examinations were performed using a commercially available second-harmonic imaging system (Toshiba XG/Artida, Toshiba Medical Systems Corporation, Otawara, Tochigi, Japan), in accordance with the recommendations of the American Society of Echocardiography and the European Association of Cardiovascular Imaging [38,39]. Imaging was conducted at two time points, three to five days after admission and at six months following MI. Left ventricular structural and functional parameters were assessed, including LV end-diastolic diameter (LVEDD), LV end-systolic diameter (LVESD), and LV ejection fraction (LVEF), as previously described [40]. LV dilatation was defined according to sex-specific thresholds (LVEDD > 56 mm in men and >51 mm in women) [41], while severe LV dilatation was defined as LVESD ≥ 46 mm in men and ≥42 mm in women [38]. Patients with LVEF < 40% were classified as having systolic dysfunction [42], and NYHA functional classes III and IV were considered indicative of advanced HF.

### 2.3. SNP Selection

Using the NCBI dbSNP database [43], SNPedia [44], GWAS Catalog [45] and lncRNASNP v3 [46], we selected variants within the MALAT1 and miR-146a gene region according to the following criteria: (i) minor allele frequency (MAF) > 10% in the non-Finish European population, based on gnomAD [47]; (ii) in silico prediction of potential functional relevance [48]; (iii) classification as expression quantitative trait loci (eQTLs) for relevant genes in the relevant organ or tissue (blood, PBMCs, arterial tissue) according to the public databases FIVEx [28] and/or GTEx [29]; (iv) previously reported GWAS associations with CVD-related traits in the GWAS Catalog [45]; (v) information on linkage disequilibrium (LD) including variants in the region, their localization within enhancers and their impact on regulatory motifs, using HaploReg v4.2 [49]; (vi) prior identification of the selected variants in published literature reporting associations with disease susceptibility. Finally, *MALAT1* rs3200401 C/T and *miR-146a* rs2910164 G/C were identified as relevant variants and were therefore selected for further analysis (Table 1).

### 2.4. Genetic Analysis

Peripheral venous blood samples were collected in EDTA tubes within three to five days following MI. Genomic DNA was extracted using the phenol/chloroform method. DNA quantity and purity were assessed using a spectrophotometric method with the NanoDrop ND-1000 (Thermo Fisher Scientific, Waltham, MA, USA). The samples were subsequently stored at −20 °C until genotyping. Gene variants *MALAT1* rs3200401 and *miR-146a* rs2910164 were detected using the TaqMan^TM^ assays for allele discrimination: C___3246069_10 and C__15946974_10, respectively, on an ABI 7500 real-time PCR system (Applied Biosystems, Foster City, CA, USA) with data acquisition and analysis performed using SDS software v1.4.0 (Applied Biosystems, Foster City, CA, USA). Each PCR reaction was prepared in a final volume of 25 µL containing 12.5 µL of TaqMan^TM^ Universal PCR Master Mix, 0.625 µL of 40× TaqMan^TM^ Genotyping Assay (including FAM- and VIC-labeled probes and primers), and approximately 120 ng of genomic DNA. The probes were labeled with a 5′ reporter dye and a 3′ quencher to enable allele-specific discrimination. PCR amplification was carried out under standard cycling conditions, including initial denaturation at 95 °C for 10 min, followed by 40 cycles of denaturation at 95 °C for 15 s and annealing/extension at 60 °C for 60 s. Samples with a genotype call rate below 95% were excluded from further analysis as part of quality control. A subset of approximately 10% of the samples was randomly selected and re-genotyped by an independent investigator. Complete concordance (100%) was observed between the repeated and initial genotyping results.

### 2.5. Isolation and Quantification of Total RNA

At the six-month follow-up, peripheral blood samples from 89 MI patients and 39 controls were collected for total RNA extraction. PBMCs were isolated using a density gradient centrifugation method with Lymphocyte Separation Medium (GE Healthcare, Chicago, IL, USA). Total RNA was extracted from freshly isolated PBMCs within 30 min of collection using TRIzol^TM^ Reagent (Invitrogen, Thermo Fisher Scientific, Waltham, MA, USA) following the manufacturer’s protocol. RNA integrity was preserved through the addition of RiboLock RNase Inhibitor (Thermo Fisher Scientific, Waltham, MA, USA), and samples were subsequently stored in RNase-free water at −80 °C until further processing. RNA quantification was performed using a BioSpec-nano spectrophotometer (Shimadzu Corporation, Kyoto, Japan), while RNA integrity was assessed through capillary electrophoresis using the RNA 6000 Nano Kit on the 2100 Bioanalyzer system (Agilent Technologies, Inc., Santa Clara, CA, USA).

### 2.6. Relative Quantification of lncRNA MALAT1

Prior to reverse transcription, 500 ng of total RNA was treated with 1 U DNase I (RNase-free; Thermo Fisher Scientific Inc., Waltham, MA, USA) at 37 °C for 30 min. The reaction was terminated by the addition of 1 µL of 25 nM EDTA followed by heat inactivation at 65 °C for 10 min. Reverse transcription was performed in a 20 µL reaction using both a random hexamer primer (Thermo Fisher Scientific Inc., Waltham, MA, USA) and an oligo(dT) primer (Thermo Fisher Scientific Inc., Waltham, MA, USA), together with RevertAid Reverse Transcriptase (Thermo Fisher Scientific Inc., Waltham, MA, USA), following the manufacturer’s instructions. The thermal cycling conditions included RNA denaturation with primers at 70 °C for 5 min (followed by immediate cooling on ice), primer annealing at 25 °C for 5 min, and enzymatic extension at 25 °C for 10 min, 42 °C for 60 min, and 72 °C for 10 min. The resulting cDNA was stored at −20 °C until further analysis. Real-time PCR was carried out in duplicate on an ABI 7500 Real-Time PCR system (Applied Biosystems, Foster City, CA, USA). Relative expression of MALAT1 was quantified using a custom TaqMan^TM^ Gene Expression Assay (Hs00273907_s1), while GAPDH was used as the reference gene and detected with a predeveloped TaqMan^TM^ assay (Hs02786624_g1; Applied Biosystems, Foster City, CA, USA).

### 2.7. Relative Quantification of miR-146a

cDNA for miRNA analysis was synthesized from total RNA using the TaqMan^TM^ MicroRNA Reverse Transcription Kit (Thermo Fisher Scientific Inc., Waltham, MA, USA), following the manufacturer’s protocol. The reverse transcription reaction was prepared in a final volume of 15 µL, containing 100 ng of RNA and a specific primer pool including TaqMan™ MicroRNA assays for miR-146a and the endogenous control RNU44 (final primer dilution 0.05×). Reverse transcription was carried out under standard conditions of 16 °C for 30 min, 42 °C for 30 min, followed by enzyme inactivation at 85 °C for 5 min and subsequent cooling at 4 °C for 10 min. The resulting cDNA was stored at −20 °C until further use. Quantitative real-time PCR was performed on an ABI 7500 Real-Time PCR System (Applied Biosystems, Foster City, CA, USA) using predeveloped TaqMan^TM^ MicroRNA assay (miR-146a assay ID 002306; RNU44 assay ID 001094). All reactions were run in duplicate under standard cycling conditions: initial denaturation at 95 °C for 10 min, followed by 40 cycles of 95 °C for 15 s and 60 °C for 1 min. Relative miR-146a expression was normalized to RNU44.

### 2.8. Quantification of COX-2 Levels

At the six-month follow-up, peripheral blood samples were collected from 89 MI patients and 39 controls. The samples were separated within 30 min of collection through centrifugation and stored at −80 °C until analysis. Plasma COX-2 concentrations were measured using the FineTest^®^ Human COX-2 (Cyclooxygenase-2) ELISA kit (Wuhan, China) according to the manufacturer’s instructions. The assay performance characteristics included a sensitivity of 0.188 ng/mL, intra-assay and inter-assay coefficients of variation of 5.98% and 5.92%, respectively, recovery range of 95% and a detection range of 0.313–20 ng/mL. Optical density was read at 450 nm using a Perkin Elmer Wallac 1420 Victor2 Microplate Reader (PerkinElmer, Inc., Waltham, MA, USA). COX-2 concentrations (ng/mL) were calculated in duplicate samples using a four-parameter logistic standard curve [50].

### 2.9. Statistical Methods

Genotype distributions and allele frequencies were estimated using the gene-counting approach. Pearson’s Chi-square (χ^2^) test was used to evaluate differences in genotype distributions and allele frequencies between controls and patients, deviations from Hardy–Weinberg equilibrium (HWE), and comparisons of categorical variables. Logistic regression was performed to estimate the strength of genetic association, with results presented as odds ratios (ORs) with corresponding 95% confidence intervals (CIs). Multivariable logistic regression analyses were performed to adjust for potential confounding factors, including age, sex, triglyceride levels, total cholesterol levels, and hypertension. The normality of continuous variables was assessed using both the Kolmogorov–Smirnov test with Lilliefors correction and the Shapiro–Wilk test. Depending on distribution, comparisons were performed using either the unpaired *t* test or the Mann–Whitney U test. Continuous variables were presented as mean ± standard deviation (SD). *p* values ≤ 0.05 were considered statistically significant. Bonferroni correction was applied for multiple comparisons where appropriate. For plasma COX-2 analyses, which involved three predefined comparisons (controls vs. patients, sex stratification, and NYHA class stratification), the adjusted significance threshold was set at *p* ≤ 0.017. For genetic association analyses, correction was applied for the two investigated variants (*MALAT1* rs3200401 and *miR-146a* rs2910164) and the two inheritance models evaluated for rs3200401 (dominant and recessive), resulting in a corrected significance threshold of *p* ≤ 0.0125. Combined allele effects were analyzed using THESIAS 3.1 software [51,52], which identifies the most frequent allele combination as the reference category and estimates associations as OR with 95% CI. Relative MALAT1 and miR-146a expression levels were normalized against the reference genes (GAPDH and RNU44, respectively) and presented as mean 2^−ΔCt^ values, for each sample. ΔCt represents the difference between Ct values of the target and reference genes. Statistical power for genetic associations was performed using the PS-Power and Sample Size calculator (v3.0.43) (Vanderbilt Department of Biostatistics, Nashville, TN, USA) [53]. Post hoc Power Calculator was used to perform power analysis for the gene expression results [54]. Multiple linear regression models were used to evaluate independent associations of MALAT1 and miR-146a expression with MI after adjustment for age, sex, and BMI, as well as the association of COX-2 levels with sex after adjustment for pharmacological therapy. Regression results were presented as β coefficients with standard error (β ± SE). Fisher’s exact test was applied for unadjusted analyses due to the low frequency of the rs3200401 TT genotype. Statistical analyses were performed using Statistica 8 (StatSoft Inc, Tulsa, OK, USA). Graphical representations were generated using GraphPad Prism v6.01 (GraphPad Software, Inc., Boston, MA, USA).

## 3. Results

### 3.1. Baseline Characteristics of the Study Population

The baseline characteristics of the control subjects and patients with first acute MI are presented in Table 2. Patients had significantly higher BMI, LDL cholesterol and triglycerides, and lower HDL cholesterol than controls. MI patients were older and had a higher proportion of males and hypertensive individuals than the control subjects, consistent with the established cardiovascular risk profile associated with MI.

### 3.2. Relative Expression of lncRNA MALAT1 and miR-146a in PBMCs from Controls and Patients Six Months After MI

**Figure 1:**
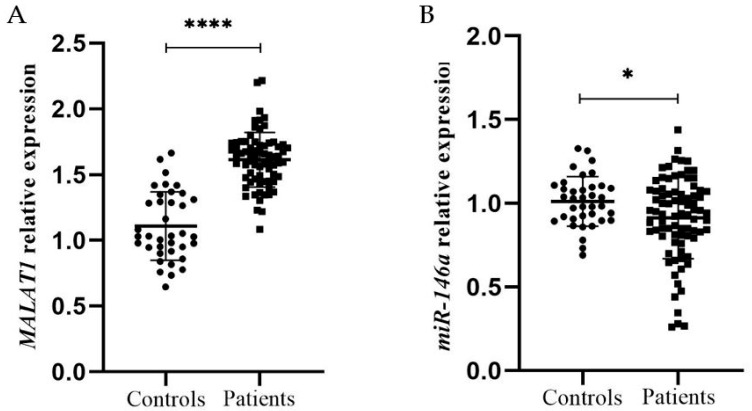
Relative expression of MALAT1 and miR-146a in PBMCs from controls and MI patients six months post-MI. Relative MALAT1 and miR-146a expression is presented as 2^−ΔCt^ values for each sample. cDNAs from PBMCs of controls (n = 39) and MI patients (n = 89) were used to quantify gene expression. The difference between the Ct values of the reference genes for MALAT1 and miR-146a, GAPDH and RNU44, respectively, and the genes of interest was used to compute the ∆Ct value. Data are presented as 2^−ΔCt^ means for both groups (controls—circles; patients—squares) ± SD. (**A**) Significant upregulation of MALAT1 was detected in PBMCs from patients compared with controls (1.614 ± 0.207 vs. 1.110 ± 0.260, respectively, *p* < 0.0001, Student’s *t* test); (**B**) significant downregulation of miR-146a was detected in PBMCs from patients compared to controls (0.913 ± 0.244 vs. 1.012 ± 0.148, respectively, *p* = 0.04, Mann–Whitney U test). * *p* ≤ 0.05, **** *p* ≤ 0.0001.

Analysis of relative lncRNA MALAT1 and miR-146a expression was conducted on PBMC samples from 89 patients six months after their first MI and 39 healthy controls. Baseline characteristics available for the expression analysis subset are presented in Supplementary Table S1. Relative expression of MALAT1 and miR-146a is presented as mean 2^−ΔCt^ values for each sample (Figure 1). MALAT1 levels were significantly upregulated in PBMCs from patients compared to controls (1.614 ± 0.207 vs. 1.110 ± 0.260, respectively, *p* < 0.0001, Student’s *t* test) (Figure 1A). The study power for this association at α = 0.05 was >90%, further validating this finding. miR-146a levels were downregulated in PBMCs from patients compared to controls (0.913 ± 0.244 vs. 1.012 ± 0.148, respectively, *p* = 0.04, Mann–Whitney U test) (Figure 1B). The observed association was supported by a study power of 80.6% at α = 0.05, suggesting adequate but borderline power for detecting the observed effect size.

In addition, we conducted multiple linear regression analysis including MALAT1 expression, age, sex and BMI. We found that MALAT1 expression remained significantly higher in patients compared to controls, independent of major confounding factors (β ± SE = 0.86 ± 0.10, *p* < 0.0001), further supporting a potential role of MALAT1 in the chronic post-MI phase. However, the association for miR-146a lost statistical significance after adjustment for confounders (β ± SE = −0.10 ± 0.14, *p* = 0.45).

In a sensitivity analysis excluding patients with T2DM, MALAT1 expression remained significantly upregulated in MI patients compared with controls (1.600 ± 0.197 vs. 1.110 ± 0.260, respectively, *p* < 0.0001, Student’s *t* test) and retained statistical significance after adjustment for age, sex, and BMI (β ± SE = 0.84 ± 0.11, *p* < 0.0001). In contrast, miR-146a expression did not reach statistical significance in this subset analysis.

### 3.3. Plasma COX-2 in Controls and Patients Six Months After MI

**Figure 2:**
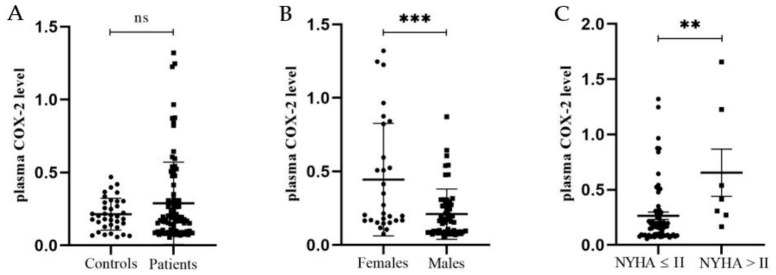
Plasma COX-2 concentrations in controls and patients six months following MI (**A**), according to sex among MI patients (**B**) and according to NYHA class six months following MI (**C**). Bonferroni correction for multiple testing was applied and *p* ≤ 0.017 was considered statistically significant. ns—not significant; ** *p* ≤ 0.01, *** *p* ≤ 0.001.

Plasma COX-2 concentrations were determined in 39 controls and 89 patients, whose PBMCs were collected six months following MI (the same individuals that were included in the expression analyses). Plasma COX-2 levels were not significantly different between controls and patients (0.214 ng/mL ± 0.109 ng/mL vs. 0.289 ng/mL ± 0.282 ng/mL, respectively, *p* = 0.88, Mann–Whitney U test) (Figure 2A). Among patients, plasma COX-2 concentrations were significantly higher in females than in males (0.444 ng/mL ± 0.382 ng/mL vs. 0.210 ng/mL ± 0.171 ng/mL, respectively, *p* = 0.001, Mann–Whitney U test) (Figure 2B). Furthermore, significantly higher plasma COX-2 levels were observed in patients with advanced HF (NYHA class III-IV) six months following MI compared with patients in NYHA class I-II (0.654 ng/mL ± 0.563 ng/mL vs. 0.264 ng/mL ± 0.274 ng/mL, respectively, *p* = 0.01, Mann–Whitney U test) (Figure 2C). The present data suggest that elevated plasma COX-2 levels may be associated with more severe functional impairment following MI.

Given the potential influence of pharmacological therapy on inflammatory signaling pathways and gene expression profiles following MI, we additionally assessed the possible influence of medication use in our cohort. The majority of patients (>90%) received standard post-MI therapy, including aspirin, clopidogrel, low-molecular-weight heparin (LMWH), nitrates, ACE inhibitors, and statins. Unfractionated heparin (UFH) was administered in 57.1% of patients in whom MALAT1, miR-146a, and COX-2 were measured, β-blockers in 80.9%, and diuretics in 14.3%. We observed significant differences in plasma COX-2 levels according to UFH and diuretic use. Therefore, finding regarding the COX-2 levels in relation to sex was additionally adjusted for UFH and diuretic therapy using multiple linear regression, and the observed association remained statistically significant (β ± SE = −0.43 ± 0.10, *p* ≤ 0.001). Because the subgroup with NYHA class > II was rather small (n = 7), adjustment for potential confounders such as therapy or sex was not feasible.

### 3.4. Association of MALAT1 rs3200401 and miR-146a rs2910164 with MI

The genotype and allele frequency distributions of *MALAT1* rs3200401 and *miR-146a* rs2910164 in controls and MI patients are shown in Table 3. and were in HWE in both controls and patients. We found a significantly higher frequency of the *MALAT1* rs3200401 TT genotype, according to the recessive model (CC + CT vs. TT), in MI patients compared to controls, with an OR of 2.58 (95% CI = 1.12–5.81, *p* = 0.02, Fisher’s exact test). Due to the low frequency of the rs3200401 TT genotype, Fisher’s exact test was applied for the unadjusted analysis. This result was adjusted for confounding factors (age, sex, triglycerides, total cholesterol and hypertension) using multiple logistic regression. The association between *MALAT1* rs3200401 and MI remained significant after adjustment, supporting an independent effect of this variant (adjusted OR (95% CI) = 4.01 (1.57–10.19), *p* = 0.003) (Table 3). The study power for this association exceeded 90% at a significance level of α = 0.0125, further supporting a potential association of *MALAT1* rs3200401 with MI susceptibility. The distributions of genotype and allele frequencies of *miR-146a* rs2910164 did not differ significantly between control subjects and MI patients.

We also examined the combined effect of the analyzed variant alleles on MI risk (Table 4). We found that the rs3200401-rs2910164 TG alleles combination was associated with a 1.57-fold increased risk of MI (TG vs. referent CG, adjusted OR = 1.57, 95% CI = 1.05–2.37, *p* = 0.03). The OR was adjusted for age, sex, triglycerides, total cholesterol and hypertension (Table 4).

### 3.5. Relative Expression of MALAT1 and miR-146a and Plasma COX-2 in Controls and Patients Six Months After MI According to MALAT1 rs3200401 and miR-146a rs2910164 Genotypes

**Figure 3:**
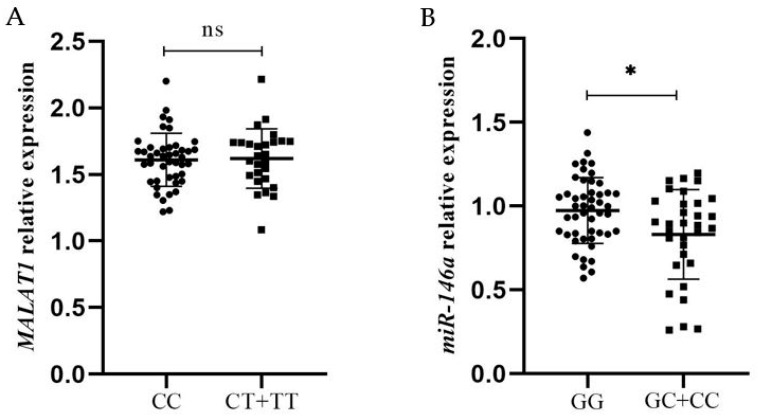
Relative expression of (**A**) MALAT1 in PBMCs of MI patients regarding the *MALAT1* rs3200401 dominant model (CC vs. CT + TT); (**B**) miR-146a in PBMCs of MI patients regarding the *miR-146a* rs2910164 dominant model (GG vs. GC + CC). ns—not significant; * *p* ≤ 0.05.

No significant differences in MALAT1 expression were observed between patients according to the *MALAT1* rs3200401 dominant model (CC vs. CT + TT: 1.610 ± 0.198 vs. 1.620 ± 0.223, *p* = 0.84, Student’s *t* test) (Figure 3A). miR-146a was downregulated in patients carrying the *miR-146a* rs2910164 C allele (GC + CC) compared to those with the GG genotype (0.832 ± 0.267 vs. 0.974 ± 0.197, respectively, *p* = 0.04, Mann–Whitney U test) (Figure 3B). Findings regarding the miR-146a expression in MI patients according to rs2910164 were adjusted for UFH therapy using multiple linear regression, as significant differences in miR-146a expression levels were observed between patients receiving and not receiving UFH. The previously observed association remained statistically significant (β ± SE = −0.27 ± 0.11, *p* = 0.02).

Plasma COX-2 concentrations were not significantly different in patients according to *MALAT1* rs3200401 (CC vs. CT + TT: 0.290 ng/mL ± 0.304 ng/mL vs. 0.284 ng/mL ± 0.252 ng/mL, respectively, *p* = 0.57, Mann–Whitney U test) or *miR-146a* rs2910164 (GG vs. GC + CC: 0.284 ng/mL ± 0.282 ng/mL vs. 0.296 ng/mL ± 0.286 ng/mL, respectively, *p* = 0.99, Mann–Whitney U test) (Supplementary Figure S1).

### 3.6. Association of MALAT1 rs3200401 and miR-146a rs2910164 with Echocardiographic Parameters Serving for Assessment of LV Function and Structure: LVEDD, LVESD and LVEF

We found that the rs3200401-rs2910164 TG allele combination was associated with early post-MI systolic dysfunction, defined as LVEF < 40% (TG vs. referent CG, adjusted OR = 1.62, 95% CI = 1.06–2.49, *p* = 0.03). The OR was adjusted for age, sex, triglycerides, total cholesterol and hypertension (Table 5). In addition, higher frequency of the *MALAT1* rs3200401-T-allele-containing genotypes was observed in patients with mildly reduced LVEF (<50%), compared to those with referent LVEF (41.85% vs. 32.02%, respectively, χ^2^ test, *p* = 0.04). We also found a higher frequency of the *MALAT1* rs3200401 TT genotype in patients with severely dilated LVESD early after MI compared to those with referent LVESD (9.59% vs. 4.04%, respectively, χ^2^ test, *p* = 0.04). No association of the investigated gene variants, individually or in allele combination, with LVEDD early after MI was observed. In addition, we found that patients carrying the *MALAT1 rs3200401* T allele had significantly higher TC levels compared to carriers of CC genotype (5.76 mmol/L ± 1.24 mmol/L vs. 5.52 mmol/L ± 1.09 mmol/L, respectively, *p* = 0.03, Mann–Whitney U test). Altogether, these findings indicate that *MALAT1* rs3200401 may be associated with both functional cardiac impairment and altered total cholesterol levels in the early post-MI phase.

## 4. Discussion

In this study, we investigated the interplay between lncRNA MALAT1, miR-146a and circulating COX-2 in the chronic phase following a first acute myocardial infarction. Six months after MI, patients demonstrated altered expression patterns characterized by upregulated MALAT1 and lower miR-146a expression compared with healthy controls, with the latter showing loss of significance after adjustment for age, sex, and BMI. We also observed a higher frequency of the *MALAT1* rs3200401 TT genotype, according to the T allele recessive model, and a higher frequency of the rs3200401-rs2910164 TG allele combination, in MI patients compared to controls, both after adjustment for confounding factors associated with MI. Furthermore, within the MI cohort, plasma COX-2 concentrations were higher in females than in males, and also in patients with advanced heart failure, defined as NYHA class III-IV, compared to those with NYHA I-II, suggesting an association between COX-2 and the severity of post-MI remodeling and HF. These findings provide new insights into long-term post-ischemic inflammatory and oxidative-stress-related molecular alterations involved in chronic post-MI remodeling.

The increased MALAT1 expression observed in post-MI patients in this study, together with lower miR-146a levels in unadjusted analyses, goes in line with the proposed mechanism in which MALAT1 may act as a ceRNA that “sponges” miR-146a. This reduces the availability of miR-146a to repress its targets, thereby diminishing its regulatory capacity over inflammatory and redox signaling. Proposed mechanism is consistent with previous reports in human endothelial cells, where MALAT1 sponging miR-146a modulates NF-κB activation and inflammatory injury [55]. Furthermore, the interaction between MALAT1 and miR-146a has been implicated in human disease contexts, as well, further supporting its biological relevance [19,56,57]. Given that miR-146a regulates inflammatory pathways by targeting TNF-α, NF-κB, TLR4 and IRAK1/TRAF6 [58], reduced miR-146a availability may contribute to a prolonged pro-inflammatory environment even months after MI. Our results are consistent with recent studies reporting increased expression of MALAT1 in PBMCs, plasma or serum of MI patients, suggesting a possible contribution to pro-inflammatory and pro-atherogenic states following MI [15,16,17,18]. However, these studies focused on the acute phase of MI, whereas our study evaluated patients six months after the event. Xue et al. observed that plasma miR-146a expression levels were increased in the early-stage of acute MI, both before and after PCI, compared to controls. However, there was a decrease in miR-146a levels after PCI compared with levels before PCI [59]. In contrast, Wang et al. demonstrated that miR-146a exerts a protective role, significantly decreasing infarct size and improving cardiac function following MIRI [60]. A recent study reported downregulated miR-146a expression in CAD patients compared to controls [61], which is in agreement with our observation of lower miR-146a expression in PBMCs from patients six months after MI and may indicate altered inflammatory regulatory pathways in the post-MI phase.

COX-2 is an inducible enzyme upregulated in response to inflammatory stimuli, oxidative stress, and tissue injury [62]. Although it has been listed among ferroptosis-associated markers in the FerrDb database [63], its involvement in ferroptosis is not specific and may reflect broader inflammatory and oxidative-stress-related processes. In the present study, plasma COX-2 levels did not differ significantly between MI patients and controls six months post-MI. However, the widespread use of statin therapy in our patient cohort represents a major confounder, as statins possess anti-inflammatory properties and have been shown to attenuate COX-2 expression in human endothelial cells [64]. This may have influenced circulating COX-2 levels in the post-MI period. Several studies show that inflammatory biomarkers, such as ICAM-1, CRP, IL-6, IL-8, TNF-α, remain elevated for months after MI compared to healthy controls, even in the stable post-MI phase [65,66,67]. However, to the best of our knowledge, no published human study has measured plasma or serum levels of COX-2 months after MI, and compared those levels to healthy controls. Furthermore, female patients exhibited significantly higher COX-2 levels than male patients, consistent with evidence suggesting sex-related differences in prostaglandin signaling pathways [68]. Importantly, we found that higher plasma COX-2 levels six months post-MI were significantly associated with higher NYHA class, suggesting a potential association with HF severity. This may reflect increased inflammatory burden and systemic oxidative stress in patients with more advanced functional impairment in the months following MI, independent of the acute ischemic event. Given that chronic HF is characterized by persistent oxidative stress and iron-dependent lipid peroxidation, COX-2 may be considered a non-specific downstream marker of inflammatory and oxidative-stress-related activity during post-infarction cardiac remodeling. A recent integrative bioinformatics analysis of human non-ischemic HF datasets identified *PTGS2* as one of the most upregulated genes in the circulating blood of patients, suggesting its potential as a diagnostic biomarker for non-ischemic HF [22]. Our findings demonstrating higher plasma COX-2 levels in patients with more severe HF are consistent with these observations.

In addition to the expression profile of the MALAT1/miR-146a/COX-2 axis, we evaluated *MALAT1* rs3200401 and *miR-146a* rs2910164, individually and in allele combination, in association with MI susceptibility, gene expression levels and plasma COX-2 concentrations in the chronic post-MI phase, and baseline echocardiographic parameters of LV structure and function. We found a significant and independent association of *MALAT1* rs3200401 rare homozygotes (TT), under the T allele recessive model, with the first acute MI. Data from the FIVEx: eQTL browser [28] show a significant increase in MALAT1 expression in peripheral blood and tibial artery by the *MALAT1* rs3200401 T allele [69]. A significantly higher frequency of the same rare T allele was observed in patients with severely dilated LVESD (TT genotype) and in those with mildly reduced LVEF (<50%) (T allele carrying genotypes), suggesting an association between the T allele and impaired LV systolic function accompanied by more pronounced LV dilatation. All the above-mentioned cardiac structural and functional characteristics have previously been identified as significant prognostic factors for HF [70]. In a recent genome-wide association study (GWAS), *MALAT1* rs3200401 T allele was associated with higher BMI [71]. The same variant was analyzed in another GWAS, for association with CAD; however, the results did not identify *MALAT1* rs3200401 as a prognostic marker for CAD [72]. Previous studies found an association between genotypes containing the rs3200401 T allele and susceptibility to MACCE, ischemic stroke and NIHSS score, i.e., disease severity, suggesting that this variant may be a biomarker for progression and poor prognosis among MI and IS patients [30,33]. In contrast, Zhu et al. found no significant association between *MALAT1* rs3200401 and IS [34]. A recent study showed that carriers of the rs3200401 TT genotype had higher total cholesterol levels in both controls and MI patients, which might contribute to atherogenesis in MI patients; however, no associations between *MALAT1* rs3200401 and susceptibility to MI were found [31]. In line with the latter study, we found that patients carrying rs3200401-T-allele-containing genotypes had significantly higher total cholesterol levels compared to CC genotype carriers. Furthermore, no significant differences were observed between controls and MI patients regarding *miR-146a* rs2910164. However, significantly lower expression of miR-146a was observed in patients carrying C-allele-containing genotypes compared with GG genotype carriers. A recent meta-analysis found that carriers of the *miR-146a* rs2910164 GG genotype had higher MI susceptibility [73], and recent studies have identified the *miR-146a* rs2910164 G allele, according to both dominant and recessive models, as a risk factor for acute MI incidence and MACE in acute coronary syndrome and MI patients [74,75,76]. Carriers of the G allele also exhibited increased inflammatory markers and oxidative stress [74]. However, the participants in these studies were of Asian ancestry (China and Pakistan) and allele frequencies in these populations differ from those observed in the Serbian population. This may explain the discrepancies between previously reported results and our findings. In addition, we examined the combined allele effect of the analyzed variants on MI risk, and found that the rs3200401-rs2910164 TG allele combination was associated with MI susceptibility, independently of age, sex, triglycerides, total cholesterol and hypertension, compared to the referent one. The same TG allele combination was associated with early post-MI systolic dysfunction, defined as LVEF < 40%.

There are certain limitations to this study that should be addressed and considered in future research. Although a reasonably large number of participants were included for genetic association analysis, the gene expression analysis was conducted with a relatively modest sample. Post hoc power analysis suggested adequate power for miR-146a; however, the observed association was borderline and not robust to adjustment for confounders, warranting validation in larger independent cohorts. Accordingly, the results regarding the effects of the investigated variants on MALAT1 and miR-146a expression and plasma COX-2 levels require confirmation in larger study populations. In addition, the patient and control groups were not fully matched for age and sex. Although multivariable analyses were performed to account for these potential confounders, residual confounding cannot be completely excluded, particularly in the analysis of plasma COX-2 levels across NYHA classes. Future studies including larger, age- and sex-matched cohorts are needed to further validate the present findings and minimize potential confounding effects. In our study, blood samples were collected at a single time point, six months after the first MI, and miR-146a and MALAT1 expression together with plasma COX-2 levels were determined from these samples. Therefore, the cross-sectional design did not allow evaluation of longitudinal changes in these molecular markers from the acute to the chronic post-MI phase. Furthermore, COX-2 was evaluated as an inflammatory and oxidative-stress-related marker, whereas canonical ferroptosis markers were not assessed. Future studies including markers such as GPX4, ACSL4, lipid peroxidation products, and iron homeostasis parameters are needed to further clarify the potential relevance of ferroptosis in chronic post-MI remodeling. Gene expression analyses were performed in PBMCs, as they represent a widely used, minimally invasive and readily accessible sample type for research and diagnostics. However, direct extrapolation of these findings to myocardial tissue should be made with caution. Future studies including cardiac tissue samples or appropriate experimental models are needed to further clarify the relationship between circulating and myocardial molecular signatures.

## 5. Conclusions

We found significant and independent upregulation of MALAT1 in patients six months after myocardial infarction. We also observed lower miR-146a expression in patients in unadjusted analyses; however, this association lost statistical significance after adjustment for confounders. *MALAT1* rs3200401 may represent a potential genetic marker associated with MI susceptibility, impaired LV systolic function, and severe LV dilatation, which could subsequently lead to poor clinical outcomes including heart failure. In addition, rs3200401-rs2910164 TG allele combination was independently associated with MI susceptibility and systolic dysfunction. Collectively, these findings support MALAT1 as a potential long-term biomarker of post-MI molecular alterations, whereas the contribution of miR-146a remains to be clarified. Further studies in larger independent cohorts are required to validate both the expression and genetic findings.

## Figures and Tables

**Table 1:** The main information on selected gene variants.

Gene	Variant (rs)	Allelic Change	Chromosome	Variant Type	MAF	eQTL (Tissues)	RegulomeDB Score	GWAS Evidence	Variants in LD (n)
*MALAT1*	rs3200401	C/T	chr11	Non-coding transcript variant	0.20	Whole blood, artery—tibial, aorta, coronary, heart—LV	1f	Yes (BMI levels)	3
*miR-146a*	rs2910164	G/C	chr5	Non-coding transcript variant	0.23	No	1f	No	5

MAF—minor allele frequency; eQTL—expression quantitative trait locus; LD—linkage disequilibrium; GWAS—genome-wide association study; LV—left ventricle; BMI—body mass index.

**Table 2:** Baseline characteristics of control subjects and patients with first acute myocardial infarction.

Variable	Controls, *n* = 381	MI Patients, *n* = 534	*p* Value
Demographic characteristics			
Sex (F/M), %	49.87/50.13	29.31/70.69	<0.01
Age, years	52.7 ± 14.3	58.4 ± 11.4	<0.01 ^#^
Clinical characteristics			
Hypertension, %	27.43	65.71	<0.01
T2DM, %	0.00	26.87	N/A
Smoking, %	55.04	64.09	ns
BMI, kg/m^2^	24.58 ± 3.74	27.26 ± 4.01	<0.01 ^#^
Systolic blood pressure, mmHg	N/A	133.25 ± 26.24	N/A
Diastolic blood pressure, mmHg	N/A	82.67 ± 15.65	N/A
Biochemical characteristics			
TC, mmol/L	5.61 ± 1.30	5.60 ± 1.15	ns ^#^
HDLC, mmol/L	1.48 ± 0.86	1.12 ± 0.34	<0.01 ^#^
LDLC, mmol/L	3.31 ± 1.23	3.67 ± 1.04	<0.01 ^#^
TG, mmol/L	1.58 ± 1.09	1.86 ± 1.27	<0.01 ^#^
CK_max_, U/L	N/A	1750.84 ± 1738.53	N/A
CK-MB_max_, U/L	N/A	158.65 ± 145.11	N/A
Tn_max_, U/L	N/A	103.48 ± 118.71	N/A
Glucose, mmol/L	N/A	8.63 ± 4.33	N/A
CRP, mg/L	N/A	24.68 ± 37.28	N/A
Post-MI discharge medications (%)			
Aspirin	N/A	99.4	N/A
Clopidogrel	N/A	98.9	N/A
UFH	N/A	64.4	N/A
LMWH	N/A	96.6	N/A
Nitrates	N/A	94.4	N/A
ACE inhibitors	N/A	94.4	N/A
Beta blockers	N/A	81.8	N/A
Diuretics	N/A	17.5	N/A
Statins	N/A	98.9	N/A
Echocardiographic characteristics			
LVEDD, mm	N/A	54.32 ± 6.10	N/A
LVESD, mm	N/A	38.97 ± 6.73	N/A
LVEF, %	N/A	45.63 ± 10.23	N/A

Values are presented as mean ± standard deviation (SD) for: age, body mass index (BMI), systolic and diastolic blood pressure, total cholesterol (TC), high-density lipoprotein cholesterol (HDLC), low-density lipoprotein cholesterol (LDLC), triglycerides (TG), peak creatine kinase level (CK_max_), peak creatine kinase–MB level (CK-MB_max_), glucose, peak troponin level (Tn_max_), C-reactive protein (CRP), left ventricular end-diastolic diameter (LVEDD), left ventricular end-systolic diameter (LVESD) and left ventricular ejection fraction (LVEF). ^#^ The Mann–Whitney U test was used to compare continuous variables with a skewed distribution between controls and MI patients. Pearson’s Chi-square (χ^2^) test was used to compare categorical variables. *p* ≤ 0.05 was considered statistically significant. T2DM—type 2 diabetes mellitus, UFH—unfractionated heparin, LMWH—low-molecular-weight heparin; ns—not significant; N/A—not applicable.

**Table 3:** Genotype frequencies of the *MALAT1* rs3200401 and *miR-146a* rs2910164 variants in MI patients and controls.

Gene	Gene Variant	Controls, *n* (%)	MI Patients, *n* (%)	OR [95% CI]	*p* Value
		*n* = 381	*n* = 534		
*MALAT1*	rs3200401 C/T				
	CC	254 (66.67)	333 (62.36)		
	CT	119 (31.23)	173 (32.40)	1.26 (0.99–1.60)	0.06
	TT	8 (2.10)	28 (5.24)		
	CC + CT	373 (97.90)	506 (94.76)	4.01 (1.57–10.19) ^#^	0.003
	TT	8 (2.10)	28 (5.24)		
	allele C/T	0.82/0.18	0.79/0.21	1.27 (0.91–1.78)	0.16
*miR-146a*	rs2910164 G/C				
	GG	232 (60.89)	317 (59.36)		
	GC	130 (34.12)	187 (35.02)	1.06 (0.85–1.34)	0.86
	CC	19 (4.99)	30 (5.62)		
	allele G/C	0.78/0.22	0.77/0.23	1.06 (0.77–1.47)	0.71

OR—odds ratio, CI—confidence interval, p—Pearson Chi-square test. Bonferroni correction was applied for the two investigated variants (*MALAT1* rs3200401 and *miR-146a* rs2910164) and the two inheritance models evaluated for rs3200401 (dominant and recessive). *p* ≤ 0.0125 was considered statistically significant. ^#^ The OR was adjusted for risk factors: age, sex, triglycerides, total cholesterol and hypertension.

**Table 4:** Combined allele effect of the *MALAT1* rs3200401 and *miR-146a* rs2910164 variants with MI risk.

Allele Combination	Frequency		OR [95% CI] ^#^	*p* Value
	Controls	MI Patients		
CG	0.6414	0.5974	referent	
CC	0.1804	0.1902	1.10 [0.77–1.58]	0.60
TG	0.1358	0.1687	1.57 [1.05–2.37]	0.03
TC	0.0425	0.0438	1.10 [0.46–2.63]	0.84

OR—odds ratio, CI—confidence interval, *p* values ≤ 0.05 were considered statistically significant. ^#^ The OR was adjusted for risk factors: age, sex, triglycerides, total cholesterol and hypertension. The order of alleles presented in the table is as follows: the first line shows the *MALAT1* rs3200401 C or T allele, and the second line shows the *miR-146a* rs2910164 G or C allele. The CG was set as the reference allele combination through the Thesias (3.1.) software.

**Table 5:** Combined effect of the *MALAT1* rs3200401 and *miR-146a* rs2910164 variants alleles with early post-MI systolic dysfunction, defined as LVEF < 40%.

Allele Combination	Frequency		OR [95% CI] ^#^	*p* Value
	LVEF > 40%	LVEF < 40%		
CG	0.6018	0.5767	referent	
CC	0.2016	0.1749	0.83 [0.55–1.27]	0.40
TG	0.1473	0.2081	1.62 [1.06–2.49]	0.03
TC	0.0493	0.0403	0.74 [0.34–1.58]	0.57

OR—odds ratio, CI—confidence interval. ^#^ The OR was adjusted for age, sex, triglycerides, total cholesterol and hypertension. The order of alleles represented in the table is as follows: the first line shows the *MALAT1* rs3200401 C or T allele, and the second line shows the *miR-146a* rs2910164 G or C allele. The CG was set as the reference allele combination through the Thesias (3.1.) software.

## Data Availability

The original contributions presented in this study are included in the article/Supplementary Materials. Further inquiries can be directed to the corresponding author.

## Supplementary Materials

The following supporting information can be downloaded at: https://www.mdpi.com/article/10.3390/biomedicines14071433/s1, Table S1: Baseline characteristics of controls and MI patients included in the expression analyses. Figure S1: Plasma COX-2 concentrations in MI patients six months post-MI regarding the (A) *MALAT1* rs3200401 and (B) *miR-146a* rs2910164.

## Abbreviations

The following abbreviations are used in this manuscript:

CVD	cardiovascular disease
MI	myocardial infarction
HF	heart failure
IS	ischemic stroke
lncRNAs	long non-coding RNAs
miRNAs	microRNAs
MALAT1	metastasis-associated lung adenocarcinoma transcript 1
STEMI	ST-elevation myocardial infarction
MACE	major adverse cardiovascular event
ceRNA	competing endogenous RNA
COX-2	cyclooxygenase-2
COPD	chronic obstructive pulmonary disease
MACCE	major adverse cardiovascular and cerebrovascular events
TC	total cholesterol
CAD	coronary artery disease
PBMC	peripheral blood mononuclear cell
PCI	percutaneous coronary intervention
T2DM	type 2 diabetes mellitus
LV	left ventricle
MMA	Military Medical Academy
LVEDD	LV end-diastolic diameter
LVESD	LV end-systolic diameter
LVEF	LV ejection fraction
HFrEF	heart failure with reduced ejection fraction
eQTLs	expression quantitative trait loci
LD	linkage disequilibrium
GWAS	genome-wide association study
BMI	body mass index
SD	standard deviation
HWE	Hardy–Weinberg equilibrium
OR	odds ratio
CI	confidence interval
HDLC	high-density lipoprotein cholesterol
LDLC	low-density lipoprotein cholesterol
TG	triglycerides
CKmax	peak creatine kinase level
CK-MBmax	peak creatine kinase–MB level
Tnmax	peak troponin level
CRP	C-reactive protein
UFH	unfractionated heparin
LMWH	low-molecular-weight heparin
ns	not significant
N/A	not applicable
ROS	reactive oxygen species
MIRI	myocardial ischemia/reperfusion injury
