# Supplementary material for: MALAT1/miR-146a/COX-2 Expression Profile Six Months After Myocardial Infarction and Association of MALAT1 rs3200401 and miR-146a rs2910164 with Disease Susceptibility
Source: Biomedicines. 2026 Jun 24;14(7):1433. doi: 10.3390/biomedicines14071433 (PMC13404195; doi:10.3390/biomedicines14071433)
Supplement: Supplementary file 1 [file biomedicines-14-01433-s001.zip › biomedicines-4357128-supplementary.pdf]

MALAT1/miR-146a/COX-2 expression profile six months after myocardial infarction and association of *MALAT1* rs3200401 and *miR-146a* rs2910164 with disease susceptibility, Macak Stefanovic et al.

Table S1. Baseline characteristics of controls and MI patients included in the expression analyses.

Variable	Controls, n=39	Patients, n=89	p
Sex, F/M, %	79.49/20.51	27.50/72.50	<0.01
Age, years	41.9±9.0	56.1±7.8	<0.01#
T2DM, %	0.00	33.78	N/A
Smoking, %	52.42	64.38	ns
BMI, kg/m^2	23.33±3.67	27.42±4.29	<0.01#
TC, mmol/L	5.55±1.34	5.63±1.19	ns#

Values are presented as mean ± standard deviation (SD) for: age, body mass index (BMI) and total cholesterol (TC). # The Mann-Whitney U test was used to compare continuous variables with a skewed distribution between controls and MI patients. Pearson's Chi-square (χ^2) test was used to compare categorical variables. p values < 0.05 were considered statistically significant. T2DM – type 2 diabetes mellitus; ns – not significant. Lipid profile data were not available for all individuals included in the expression-analysis subset.

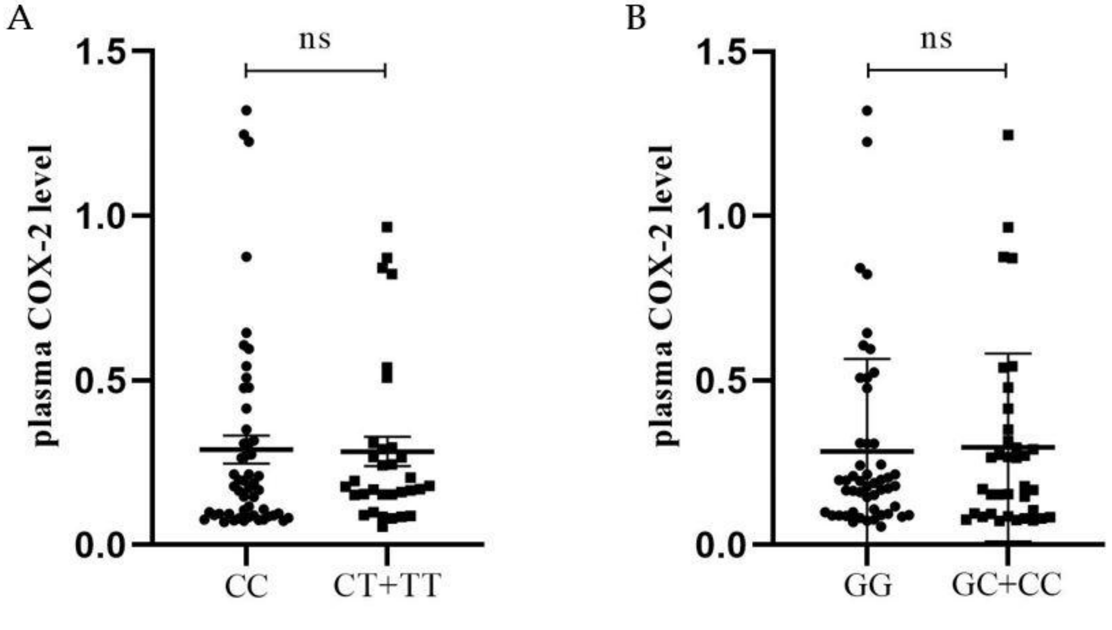

Figure S1. Plasma COX-2 concentrations in MI patients six months post-MI regarding the (A) *MALAT1* rs3200401, and (B) *miR-146a* rs2910164. Plasma COX-2 was not significantly different patients according to *MALAT1* rs3200401 dominant model (CC vs. CT+TT: 0.290 ng/mL ± 0.304 ng/mL vs. 0.284 ng/mL ± 0.252 ng/mL, respectively, p=0.57, Mann-Whitney U test). Plasma COX-2 was not significantly different according to *miR-146a* rs2910164 dominant model (GG vs. GC+CC: 0.284 ng/mL ± 0.282 ng/mL vs. 0.296 ng/mL ± 0.286 ng/mL, respectively, p=0.99, Mann-Whitney U test).
